# Bioprosthetic Valves for Lifetime Management of Aortic Stenosis: Pearls and Pitfalls

**DOI:** 10.3390/jcm12227063

**Published:** 2023-11-13

**Authors:** Konstantinos S. Mylonas, Dimitrios C. Angouras

**Affiliations:** 1 Department of Cardiac Surgery, Onassis Cardiac Surgery Center, 17674 Athens, Greece; 2 Department of Cardiac Surgery, Attikon University Hospital, School of Medicine, National and Kapodistrian University of Athens, 15772 Athens, Greece; dangouras@yahoo.com

**Keywords:** biological prosthesis, surgical aortic valve replacement, transcatheter aortic valve replacement, Resilia, Intuity, Perceveal, Ross procedure, Ozaki technique, lifetime management, aortic stenosis

## Abstract

This review explores the use of bioprosthetic valves for the lifetime management of patients with aortic stenosis, considering recent advancements in surgical (SAV) and transcatheter bioprostheses (TAV). We examine the strengths and challenges of each approach and their long-term implications. We highlight differences among surgical bioprostheses regarding durability and consider novel surgical valves such as the Inspiris Resilia, Intuity rapid deployment, and Perceval sutureless bioprostheses. The impact of hemodynamics on the performance and durability of these prostheses is discussed, as well as the benefits and considerations of aortic root enlargement during Surgical Aortic Valve Replacement (SAVR). Alternative surgical methods like the Ross procedure and the Ozaki technique are also considered. Addressing bioprosthesis failure, we compare TAV-in-SAV with redo SAVR. Challenges with TAVR, such as TAV explantation and considerations for coronary circulation, are outlined. Finally, we explore the potential challenges and limitations of several clinical strategies, including the TAVR-first approach, in the context of aortic stenosis lifetime management. This concise review provides a snapshot of the current landscape in aortic bioprostheses for physicians and surgeons.

## 1. Background

Surgical aortic valve replacement (SAVR) is considered the gold standard of care for patients with severe aortic stenosis (AS). Although the long-term durability of mechanical prostheses is indisputable, these valves require life-long anticoagulation with vitamin K antagonists (VKAs), predisposing patients to both thromboembolic and hemorrhagic events. Therefore, an increasing number of young, active patients, including women of childbearing age, are now opting for bioprosthetic valve implantation [1].

Undoubtedly, transcatheter aortic valve replacement (TAVR) has revolutionized the treatment paradigm for older and frail patients with severe AS [2]. The midterm outcomes of transcatheter aortic valves (TAVs) have been encouraging and, in certain aspects, non-inferior to surgery. As a result, TAVR procedures are also gaining popularity among low-risk and young patients [3].

In this ever-changing landscape, Heart Teams face an unprecedented challenge in devising a lifetime management plan for patients with AS opting for an aortic bioprosthesis. An optimal “shared decision-making” process should involve a comprehensive discussion with the patient, considering evidence-based information on available options, the provider’s expertise, and the patient’s values and preferences. Each method’s significant limitations or knowledge gaps must be fully disclosed to the patient to avoid limited engagement and a lack of understanding, which may eventually lead to distrust [4]. This editorial addresses critical, relevant questions frequently asked by patients and debated among cardiovascular providers.

## 2. Surgical Aortic Valve Replacement Using Biologic Prostheses: Starting on the Right Foot

### 2.1. Surgical Bioprostheses Are Not All Equal

A wide range of surgically implanted bioprostheses are currently available. They can be classified into (a) porcine or pericardial, based on the origin of the tissue—either porcine aortic valves or bovine pericardium, respectively—and (b) stented or stentless, depending on the presence or absence of a stent frame that includes a circular or scallop-shaped external ring used for attaching the prosthesis to the aortic annulus. Stented pericardial aortic bioprostheses are further subdivided based on their design into internally or externally mounted, depending on whether their bovine pericardial leaflets are internally sewn or externally affixed onto the three stent posts of the frame [5].

Surgical aortic bioprostheses (SAVs) manifest disparities in terms of implantability, hemodynamic efficacy, durability, and appropriateness for potential Valve-in-Valve scenarios. These disparities may stem from variations in tissue provenance, processing methodologies, and valve design, underscoring the imperative for thorough contemplation within the context of the lifetime management of AS.

The Carpentier-Edwards Perimount (CE-P) valve, an internally mounted stented pericardial valve, stands as the most extensively researched bioprosthesis concerning its long-term outcomes, even encompassing individuals below the age of 60. Bourguignon et al., in their study involving 2659 patients who underwent SAVR with the CE-P valve, demonstrated age-stratified freedom from reoperation due to structural valve deterioration (SVD) at 15 and 20 years of 70.8 ± 4.1% and 38.1 ± 5.6%, respectively, for the under-60 group, 82.7 ± 2.9% and 59.6 ± 7.6% for those aged 60 to 70 years, and a remarkable 98.1 ± 0.8% at 15 years and beyond for the eldest cohort [6]. The expected valve durability for the entire cohort was estimated at 19.7 years. In a study of 522 relatively young patients (ages 50–65) who received the CE-P bioprosthesis, the actuarial freedom from reoperation rates due to SVD at 10, 15, and 20 years was 91 ± 2, 76 ± 3, and 50 ± 6%, respectively [7]. After employing competing risk analysis, the actual risk of explantation secondary to SVD at 20 years was 30 ± 3%, and the overall projected valve durability for this specific age group was 19 years. These metrics may serve as a benchmark for both established and emerging valves, including TAVs.

Several other surgical bioprostheses have demonstrated inferior durability. The Toronto SPV (T-SPV) bioprosthesis, a stentless porcine aortic valve approved by the Food and Drug Administration (FDA) for clinical use in the United States in 1997, began showing signs of significantly elevated SVD rates and subsequent reoperation as early as the seventh year after implantation. When analyzing a cohort of 357 patients, with a mean age of 65 ± 10 years, who underwent SAVR with the T-SPV bioprosthesis, David et al. revealed a 12-year freedom from SVD rate of 69 ± 4% for the entire cohort, dropping to 52 ± 8% for patients below 65 years of age [8]. This outcome fell below optimal standards, ultimately leading to the discontinuation of the T-SPV from the market.

Numerous reports have emerged, highlighting the suboptimal durability of the Sorin Mitroflow and Abbott (St. Jude Medical) Trifecta aortic bioprostheses, both externally mounted stented pericardial valves [9,10,11,12,13]. Lam et al., after analyzing 2004 biological SAVR procedures encompassing 923 Carpentier-Edwards Magna Ease (CE-ME) (the evolutionary iteration of the CE-P valve), 719 Trifecta, and 362 Mitroflow bioprostheses over a mean follow-up duration of 4.1 ± 2.4 years, demonstrated that the freedom from reintervention after implantation of the CE-ME bioprosthesis was significantly higher compared to both the Trifecta and Mitroflow bioprostheses [10]. Cox regression analysis revealed that the Trifecta (HR, 6.3; 95% CI, 2.6–15.2; and *p* < 0.0001) and Mitroflow (HR, 6.0, 95% CI, 2.4–15.1; *p* < 0.0001) valves were associated with diminished event-free survival. Cangut et al., in a 10-year follow-up of a randomized trial, established a significantly higher late risk of reinterventions for the Mitroflow group (22%) compared to the CE-ME (0%; *p* < 0.001) group and the Abbott (St. Jude Medical) Epic (5%; *p* = 0.008) group [11].

Similarly, in a network meta-analysis involving 31,029 SAVR patients from ten studies, the Trifecta valve exhibited significantly elevated rates of SVD-related reinterventions in comparison to the CE-ME, albeit not the Mitroflow valve. The CE-ME (incident rate ratio, 0.18; 95% CI, 0.07–0.47) and CE-P (incident rate ratio, 0.34; 95% CI, 0.12–0.98) were associated with a significantly lower rate of all-cause reintervention when compared with the Trifecta valve [12]. Another meta-analysis incorporating 15 observational studies with a total of 23,539 patients who underwent SAVR with externally mounted (Trifecta and Mitroflow) or internally mounted (CE-P) bioprostheses showed that externally mounted valves were linked with higher reoperation rates for SVD (HR, 3.55; 95% CI 2.67–4.72; and *p* < 0.001) and for any cause (HR 9.36, 95% CI 3.70–23.67; *p* < 0.001). Furthermore, externally mounted valves exhibited elevated all-cause mortalities (HR 1.33, 95% CI 1.13–1.56; *p* < 0.001) [13]. In vitro accelerated wear testing revealed the inferior mechanical durability of externally wrapped pericardial valves, primarily attributed to marked mechanical abrasion at the commissural region [14].

Moreover, both the Trifecta and Mitroflow valves, aside from their elevated risk for early SVD, present additional disadvantages concerning prospective Valve-in-Valve (ViV) procedures. Their design and proximity of their leaflets to the coronary ostia following TAV deployment result in an augmented likelihood of coronary obstruction. A multivariable analysis of data from the Valve-in-Valve International Data (VIVID) Registry revealed a sevenfold increase in the risk of coronary obstruction with externally mounted stented or stentless bioprostheses (OR, 7.67; 95% CI, 3.14–18.7; and *p* < 0.001) [15].

These weaknesses above have exerted substantial repercussions. The Mitroflow valve has been discontinued and supplanted with the Crown PRT (Corcym, London, UK), an externally mounted pericardial valve featuring “Phospholipid Reduction Treatment—PRT”, designed to mitigate phospholipid content in pericardial tissue and consequently reduce calcium uptake. Similarly, Abbott opted to discontinue the sale and distribution of Trifecta valves in the United States as of 31 July 2023 [16].

The disparities in durability among surgical bioprostheses necessitate meticulous consideration, especially when juxtaposed with transcatheter bioprostheses. In the NOTION (Nordic Aortic Valve Intervention) trial, 280 all-comer patients with severe AS were randomized to undergo either TAVR (*n* = 145) or SAVR (*n* = 135) [17]. The patients had a low surgical risk (mean STS score 3.0 ± 1.7%), despite a mean age of 79.1 ± 4.8 years. Over an 8-year follow-up period, the estimated risks for all-cause mortality, stroke, myocardial infarction, or their composite outcome were comparable between TAVR and SAVR. The risk of bioprosthetic valve failure was also similar (8.7% vs. 10.5%; *p* = 0.61), yet the risk of SVD was lower following TAVR than SAVR (13.9% vs. 28.3%; *p* = 0.0017). While this finding has sparked enthusiasm in the realm of TAVR, it is important to note that the SAVR arm of the trial included patients who were implanted with the Trifecta and Mitroflow valves at rates of 24% and 10%, respectively, while only 10% of patients received a CE-P valve. In view of the durability issues presented above, prudent caution is warranted when interpreting these results, as slightly less than 40% of SAVR procedures were performed using bioprostheses that are now both withdrawn from the market due to early SVD.

### 2.2. New Generation Biological Prostheses

#### 2.2.1. Inspiris Resilia Aortic Valve

Edwards Lifesciences (Irvine, CA, USA) developed the Resilia tissue technology to address the issue of SVD caused by the calcification of the bovine pericardium. The technology aims to mitigate calcification by (a) introducing a more stable and irreversible capping technology to eliminate free glutaraldehyde residuals and prevent calcium binding and (b) glycerolizing the tissue to modify its water content, enabling dry, glutaraldehyde-free storage without the need for preoperative rinsing of the valve [18]. The commercially available Inspiris Resilia aortic bioprosthesis, based on the CE-ME frame, incorporates two novel features designed for potential future ViV procedures: (a) fluoroscopically visible size markers integrated into valve sizes ranging from 19 to 25 mm and (b) a specifically designed stent frame, referred to as Vfit technology, that enables controlled and predictable expansion of the valve’s internal orifice during TAV deployment. This innovation allows for the implantation of a larger valve while obviating the need for a high-pressure bioprosthetic valve fracture. A successful ViV-TAVR procedure within a Resilia valve has already been reported [19].

The multicenter COMMENCE trial reported on the five-year USA experience with nearly 700 patients who received a Resilia valve [20]. The mean patient age was 66.9 ± 11.6 years. The trial demonstrated promising results, with 5-year actuarial freedom from all-cause mortality, SVD, and all-cause reintervention at 89.2%, 100%, and 98.7%, respectively. At five years, the EOA was 1.6 ± 0.5 cm^2^, the mean gradient was 11.5 ± 6.0 mm Hg, and 97.8% of patients were classified as NYHA class I/II. Furthermore, 97.8% and 96.3% of patients showed none/trace paravalvular and transvalvular regurgitation, respectively.

#### 2.2.2. Rapid Deployment Bioprosthesis (Intuity Elite)

The Intuity Elite valve (Edwards Lifesciences, Irvine, CA, USA), also referred to as a rapid deployment valve, is a stented bioprosthesis made from bovine pericardium and built upon the CE-P valve platform [21]. It incorporates an expandable stainless steel, cloth-covered frame on the inflow side, allowing rapid implantation without the need for traditional suturing to attach it to the aortic annulus. Fixation is achieved by placing three equally spaced, non-pledgeted, braided sutures through the aortic annulus at the nadir of each cusp. After proper positioning on the annulus, snares or tourniquets are employed to secure the valve. The inflow frame of the valve is expanded under specific inflation pressures with a balloon catheter placed in a predefined position within the delivery system [21].

This novel bioprosthesis maintains the advantages of conventional surgical bioprosthetic valves, such as low rates of paravalvular leaks and anticipated long-term durability. Additionally, it introduces significant enhancements, including improved implantability to minimize cardiopulmonary bypass and myocardial ischemia times and facilitate minimal access approaches [22]. Furthermore, the Intuity Elite valve exhibits an improved hemodynamic profile, making it competitive with TAVs [23,24,25]. This enhanced hemodynamic performance can be attributed to the subvalvular stent-based fixation system, which reshapes and widens the left ventricular outflow tract (LVOT) and, together with the absence of any pledget material, reduces turbulent flow within the LVOT [26]. On the other hand, the subvalvular frame expansion appears to be etiologically associated with an increased rate of permanent pacemaker implantation (PPI). Lazkani et al. analyzed 30 studies involving 3993 patients and reported an overall PPI prevalence of 8.6% at discharge following a rapid deployment SAVR [27]. Notably, reported PPI rates vary considerably among studies and are influenced by conservative versus aggressive PPI strategies, as conduction abnormalities often recover within several days [27].

Pelce et al. conducted a 5-year outcome analysis of the Intuity valve, reporting a 97% freedom from reoperation, a 4.1% stroke rate per patient-year, and an estimated cardiovascular mortality of less than 5% [28]. In this series, the PPI rate for new-onset conduction disturbances was 3.5% [21,28]. A recent systematic review of 45 clinical studies involving 12,714 patients found that the incidence of paravalvular leaks following Intuity deployment ranged from 0.24% to 0.70%. Stroke (2.2%) and myocardial infarction (0%) rates were also low. Notably, hospital costs ranged from USD 37,187 to USD 44,368 for Intuity compared to USD 69,389 for TAVR. Thirty-day mortality for the Intuity valve was 3.8%, while long-term mortality spanned between 2.6% and 20% [29].

#### 2.2.3. Sutureless Bioprosthesis (Perceval)

The Perceval bioprosthesis (Corcym, London, UK), also known as the sutureless valve, represents another innovative aortic surgical bioprosthesis made from bovine pericardium. Its primary purpose is to facilitate rapid, secure, and effective implantation through the utilization of modern deployment techniques. The valve is constructed upon a self-expanding and elastic nitinol alloy stent, consisting of two rings and nine connecting struts, engineered to provide structural support to the valve and secure its positioning without the need for permanent sutures. The stent design mimics the anatomy of the aortic root and, owing to its flexibility, accommodates the dynamic movements of the aorta, thereby reducing stress on the leaflets.

Much like the Intuity valve, the Perceval bioprosthesis is correlated with reduced operative times and streamlines minimal access procedures. In direct comparison, these two valves exhibited comparable in-hospital mortality rates and rates of major postoperative complications. The Perceval valve demonstrated shorter cross-clamp and cardiopulmonary bypass durations, along with increased utilization of an anterior right thoracotomy for access. Conversely, the Intuity valve was associated with a significantly superior hemodynamic profile and a reduced incidence of postoperative mild aortic regurgitation compared to the Perceval valve [30].

The largest real-world study on the Perceval valve analyzed a total of 1652 patients, with a mean age of 75 years and an average EuroSCORE II of 4.1 (±6.3) [31]. A minimally invasive approach was utilized in approximately half of these patients. The incidence of transient ischemic attacks, disabling strokes, and non-disabling strokes was minimal, at 0.4%, 0.4%, and 0.7%, respectively. PPI was required for 5.7% of patients. Central regurgitation ≥2 was observed in 0.2% of cases, while paravalvular leak ≥2 was observed in only 0.1%. At a maximum follow-up period of 8 years, cardiovascular deaths accounted for 1.9% of cases, and valve-related reintervention was required in 0.8% of cases. In a recent study involving 784 patients, Lamberigts et al. analyzed their 13-year experience with the Perceval valve, boasting the longest follow-up period to date, and reported a 10-year freedom from the reintervention rate of 94% [32].

### 2.3. The Impact of Hemodynamics on the Durability and Performance of Biological Prostheses

Optimizing prosthesis size is a cardinal aspect of aortic valve replacement. The term patient–prosthesis mismatch (PPM) is used to describe the discrepancy between the effective orifice area (EOA) of an implanted prosthesis and the EOA that is dictated by patient somatometrics. The mathematical expression of this dogma was introduced in the form of the indexed EOA (iEOA), also known as the ratio of EOA over patient body surface area (BSA). As such, severe PPM is defined by an iEOA of less than <0.65 cm^2^/m^2^, while moderate mismatch is seen between 0.65 and 0.85 cm^2^/m^2^ [33].

The impact of PPM on postoperative outcomes has been heavily debated. In a recent analysis of the Society of Thoracic Surgeons (STS) database, including nearly 60,000 patients, increased mortality and redo SAVR rates due to SVD were noted in the setting of PPM, irrespective of severity [34]. A meta-analysis of over 108,000 patients also showed that PPM of any degree decreases five- and ten-year survival [33].

Although randomized controlled trials have generally shown equal or better hemodynamic performance of TAVs compared with surgical valves, the incidence of moderate and severe PPM after TAVR is considerable, estimated at 25–43% and 6.5–12%, respectively [35]. The most extensive study to date, involving 62,125 patients from the STS/ACC TVT registry, reported a 40% incidence of severe PPM in patients who received a small (≤23 mm diameter) TAV [35]. Severe PPM, though not moderate PPM, following TAVR has also been identified as an independent predictor of increased all-cause cardiovascular mortality [35]. Notably, the incidence of PPM appears to be higher with later-generation TAVs due to the development of a skirt covering to mitigate paravalvular regurgitation, which reduces the EOA [36].

Interestingly, the hemodynamic performance of bioprostheses also impacts their durability. An extensive series of 12,569 SAVR patients found that PPM and higher postoperative peak and mean aortic valve gradients were significantly associated with explant for SVD [37]. The effect of the gradient on SVD was particularly pronounced in patients younger than 60 years. Similarly, a meta-analysis of 29 observational studies, comprising a total of 25,490 patients, revealed PPM to be a significant risk factor for SVD (hazard ratio [HR] 1.95, 95% confidence interval [CI] 1.56–2.43, and *p* < 0.001) over a mean follow-up period of 18.5 years [38]. Considering these data, avoiding PPM during the index intervention appears to be crucial in the lifetime management of AS.

### 2.4. Liberal Aortic Root Enlargement during the Index SAVR

Opting for a liberal approach to treating a small aortic annulus during the original surgery may hold the key to preventing PPM. Not surprisingly, aortic root enlargement (ARE) procedures are more frequently indicated in women, as they are more likely to have smaller aortic annuli [39]. From a technical perspective, an anterior enlargement can be achieved using a Konno-type incision towards the right ventricular outflow (a less common technique in adult cardiac surgery), while a posterior enlargement can be accomplished through a Nicks or Manougian procedure towards the mitral annulus [40]. Additionally, the emerging Yang technique can be employed to enlarge the aortic root by 3–4 sizes, involving a Y incision at the aortomitral curtain followed by the placement of a rectangular patch [41]. Despite initial skepticism, ARE procedures in the setting of isolated SAVR do not seem to influence perioperative mortality, long-term survival, or postoperative complication rates, including reoperation for bleeding, myocardial infarction, stroke, or complete heart block/permanent pacemaker needs [39,42,43].

On the other hand, suboptimal outcomes have been reported following a TAV-in-SAV procedure in patients who received a small bioprosthesis during the initial SAVR, leading to PPM [44,45]. Consequently, with the increasing popularity of TAV-in-SAV procedures, there has been a rise in the rate of ARE procedures (from 3.9% to 6.3%), resulting in a notable decline in the incidence of PPM from 23% to 16% [46]. These trends underscore the lasting impact of ARE procedures and are expected to strengthen further in the future.

### 2.5. Alternative Surgical Options

#### 2.5.1. The Ross Procedure

The Ross procedure has also been gaining traction as an alternative for young and middle-aged adult patients. A recent meta-analysis of reconstructed patient-level data associated the Ross procedure with lower all-cause mortality compared to mechanical SAVR (despite a higher risk of reoperations) [47]. Autograft wrapping using remnant aortic wall and/or Vicryl^®^ mesh results in a 74% reduction in the risk of autograft reintervention due to neoaortic root dilatation [48].

In expert hands, the Ross procedure has an early mortality rate of less than 1% [49,50]. The largest European series exploring Ross procedures in adults analyzed 2444 patients with a mean age of 44.1 ± 11.7 years. Major hemorrhagic events, valve thrombosis, stroke, and endocarditis occurred with an incidence of 0.15%, 0.07%, 0.13%, and 0.36% per patient-year, respectively [49]. The risk for autograft and right ventricular outflow tract reintervention was also low (0.69% and 0.62% per patient-year, respectively). In Tirone David’s series, 20-year freedom from any operation, reintervention on the pulmonary autograft, and the pulmonary homograft was 83.2%, 88.5%, and 91.8%, respectively [51]. Of note, estimated survival after 10 (95.1% [50]), 20 (89.2% [51]), and 25 years (75.8% [49]) does not seem to differ statistically from the general population.

#### 2.5.2. The Ozaki Technique

Professor Ozaki utilized glutaraldehyde-treated autologous pericardium to perform a trileaflet aortic valve neocuspidization (AVneo). In 2011, the Tokyo-based group published their initial experience with 404 patients, who postoperatively demonstrated favorable hemodynamics and low rates of aortic valve insufficiency (AI) [52].

Proponents of the Ozaki procedure have described several benefits in terms of physiology. To begin with, cusp reconstruction preserves the natural expansion of the aortic root during systole. In contrast to classic ring annuloplasty repairs [53], AVneo does not require the use of a sewing ring. Therefore, it achieves maximum EOA and low transvalvular gradients. Lastly, long-term coumadin anticoagulation is not required. In 2018, the Ozaki group described their full cohort of 850 patients, in whom overall survival, freedom from reoperation, and the rate of moderate or greater AI were 85.9%, 95.8%, and 7.3%, respectively, within a mean follow-up of 4.4 years [54]. Long-term data are warranted to define the role of the Ozaki technique in the armamentarium against aortic valve pathology.

## 3. Surgical Bioprosthesis Failure: Now What? TAVR-in-SAVR vs. Redo SAVR

Synthesizing data from 12 individual studies, a 2021 meta-analysis compared 8048 patients undergoing ViV-TAVR to 8159 patients who were treated with redo SAVR [55]. The study found no significant differences in the perioperative rates of stroke, myocardial infarction, acute kidney injury requiring dialysis, major vascular complications, paravalvular leak, PPI, or 30-day readmission between the two treatment modalities. However, the transcatheter intervention was associated with lower rates of 30-day mortality (random-effects model: odds ratio [OR]—0.52; 95% confidence interval [CI]—0.39 to 0.68; and *p* < 0.001) and major bleeding, as well as a shorter hospital stay. On the other hand, TAVR-in-SAVR was associated with significantly higher rates of severe post-procedural PPM compared to surgical intervention (OR: 4.63; 95% CI: 3.05–7.03, and *p* < 0.001) [55]. Notably, PPM following TAVR-in-SAVR is an independent risk factor for future reinterventions and inferior long-term survival [56]. As a result, the early mortality benefit of TAVR-in-SAVR compared to redo SAVR was lost at one year (random-effects model: OR—0.90; 95% CI—0.61 to 1.32; and *p* = 0.545), suggesting that redo SAVR might provide better survival in the long run [55,56].

In accordance with the above, an analysis of 717 propensity score-matched pairs of TAVR-in-SAVR versus redo SAVR from a large, nationwide, administrative French database demonstrated lower rates of the composite endpoint of all-cause mortality, all-cause stroke, myocardial infarction, and major or life-threatening bleeding at 30 days following a transcatheter approach. However, in the long term, no significant difference was observed between the two groups regarding the composite endpoint encompassing cardiovascular death, all-cause stroke, myocardial infarction, and rehospitalization for heart failure. Notably, after approximately 1.5 years, the incidence curves crossed and rapidly diverged to a follow-up of 4 years, favoring Redo SAVR [57].

Therefore, it can be concluded that while TAVR-in-SAVR remains a valuable treatment option for degenerated aortic bioprostheses in older and frail patients with high operative risk, redo SAVR may be the preferred choice for younger patients with low surgical risk and a longer life expectancy, particularly those with small-sized surgical bioprostheses, to mitigate the long-term complications associated with post-procedural PPM. Other essential factors that need to be considered include (a) whether surgical bioprostheses have an expandable frame or a ring amenable to balloon fracture and (b) whether they are associated with a high risk of coronary obstruction during the TAVR-in-SAVR procedure or difficulty in coronary access over time (see Section 4.2).

## 4. TAVR Challenges

### 4.1. TAV Explantation: A Rare, Yet Prominent Issue—Is TAV-in-TAV Preferable?

The frequency of TAV explantation has been estimated at approximately 0.5%, with the number of explants continuously increasing [58]. The median time to explantation ranges from 4 to 11 months [58,59]. Interestingly, one in four patients was deemed low surgical risk at the time of the index TAVR intervention [59]. Common indications for TAV explantation include procedure-related failure, endocarditis, paravalvular leak, SVD, and PPM. Alarmingly, surgery has to be performed on an emergent or urgent basis in more than half of the cases [59]. Overall, 55–65% of patients need to undergo concomitant procedures, including aortic root repair/replacement in 13–33% of the cases [58]. The latter occurs due to the incorporation of the TAV stent frame into the aortic wall over time, often requiring extensive dissection to explant. Reported operative mortality is remarkably high, i.e., 13% to 20% [58,60,61].

On the other hand, where surgery for prosthetic valve endocarditis (PVE) is concerned, TAVs do not appear to be associated with worse outcomes. In a recent analysis from the STS database, including 374 TAVR and 5883 SAVR patients who were operated on for active PVE, the TAVR group was associated with fewer aortic root abscesses compared to SAVR (13.4% versus 21.3%, *p* < 0.001), received less aortic root repair (24.9 vs. 34.8%; *p* < 0.001), and had shorter cardiopulmonary bypass/aortic cross-clamp times. Following risk adjustment, previous TAVR itself was not associated with operative mortality (odds ratio 0.99; 95% confidence interval 0.72–1.38) [62].

In selected cases of TAV dysfunction, a TAV-in-TAV strategy may be considered. The frequency of Redo TAVR among TAVR patients has been reported to be between 0.33 and 0.46% [63]. Indications for Redo TAVR include TAV stenosis, regurgitation, or combined mixed disease. Clearly, Redo TAVR is not an option for endocarditis. Device success rates in the international Redo TAVR registry were 85.1%, with most failures attributed to high residual gradients or regurgitation [64]. When compared with a matched group undergoing TAV explantation in a report from the Centers for Medicare and Medicaid Services, repeat TAVR was found to be associated with lower 30-day mortality (6.2% vs. 12.3%; *p* = 0.05) and fewer major adverse cardiovascular events (RR for THV explantation: 2.92; 95% CI: 1.88–4.99; and *p* ≤ 0.001). However, one-year mortality rates were similar for both approaches (21.0% vs. 20.8%; *p* = 1.000) [64].

The recent first report of the EXPLANTORREDO-TAVR global registry offered new insight into the TAV explantation versus TAV-in-TAV conundrum [65]. The incidence of reintervention after TAV failure was 0.59%, with an increasing volume during the study period. Median time from index-TAVR to reintervention was shorter in TAV-explant vs. Redo TAVR (17.6 months vs. 45.7 months; *p* < 0.001), respectively. TAV-explant had more PPM (17.1% vs. 0.5%; *p* < 0.001) as an indication for reintervention. This is reasonable, as a TAV-in-TAV could only be expected to further aggravate the poor hemodynamic profile of these valves. Conversely, Redo TAVR was more common in cases of SVD (63.7% vs. 51.9%; *p* = 0.023), with a similar incidence of ≥ moderate paravalvular leak between groups (28.7% in TAV explanation vs. 32.8% in Redo TAVR; *p* = 0.44). On follow-up, compared with Redo TAVR, TAV-explant had higher mortality at 30 days (13.6% vs. 3.4%; *p* < 0.001) and 1 year (32.4% vs. 15.4%; *p* = 0.001), but on landmark analysis, mortality was similar between groups after 30 days.

### 4.2. TAV-in-SAV and TAV-in-TAV: When Is the Coronary Circulation in Jeopardy?

A subset of patients may not be suitable candidates for ViV interventions due to anatomic limitations related to coronary ostia. A coronary obstruction can occur after an index TAVR procedure, as attempts are made to implant TAVs higher in relation to the aortic valve annulus to reduce the risk of conduction disturbances. However, TAV-in-SAV procedures (TAVR-after-SAVR) are associated with a four to six times higher risk of coronary obstruction (2.5–3.5%) compared to TAVRs on native valves [66]. This results from the fact that during TAV-in-SAV procedures, the leaflets of the initial surgical bioprosthesis are anchored in the open position, which, in the setting of low coronary heights or inadequate sinuses, predisposes to sinus sequestration or direct obstruction at the coronary ostium [67]. The risk is higher in the case of TAVRs on stentless surgical bioprostheses or on stented bioprostheses with externally mounted leaflets, as outlined in Section 2.1.

Coronary ostium obstruction can also occur after TAV-in-TAV (Redo TAVR), particularly when a self-expandable TAV was used in the index procedure. Cedars Sinai has published one of the largest single-center datasets on Redo TAVR. The authors found that 45.5% in the Evolut R/Evolut PRO group and 2.0% in the SAPIEN 3 group had a CT-identified risk of sinus sequestration at one or both coronary arteries. TAVR was classified as high-risk when (1) the prior TAV commissure level was above the sinotubular junction (STJ) and (2) the distance between TAV and STJ was <2.0 mm in each coronary sinus. These findings further support the role of CT screening in Redo TAVR candidates (especially in the setting of low STJ height) [68].

Bioprosthetic scallop intentional laceration during ViV (BASILICA procedure) is a transcatheter technique performed prior to the TAV implantation to prevent iatrogenic coronary artery obstruction. During this procedure, the operator employs an electrified wire to split the bioprosthetic leaflets to prevent coronary occlusion. In the original BASILICA series, the primary success rate and freedom from coronary obstruction and reintervention were 93% and 100%, respectively [69]. In cases where inadequate leaflet “splay” following traditional BASILICA is expected based on pre-procedural analysis (heavily calcified leaflets, very small valve-to-coronary distance, restriction from the outer frame of a TAV in a TAV-in-TAV setting), the modified balloon-assisted BASILICA procedure may be indicated to set the groundwork for a safe TAV implantation [70].

### 4.3. Challenging Access for Future Coronary Angiography and Percutaneous Coronary Intervention Following TAVR

Even if a ViV procedure is not required, obtaining coronary access for angiography and potential percutaneous coronary intervention following TAVR can be challenging. The prevalence of coronary artery disease (CAD) in the TAVR population has been approximately 50%, with about half of those with CAD exhibiting multivessel disease [71]. Among 142,845 Medicare patients treated with TAVR from 2012 to 2017, 4.7% were admitted with acute coronary syndrome (ACS), mostly non-ST-segment elevation myocardial infarction (NSTEMI), after a median time of only 297 days post-TAVR, with 48% of admissions occurring within 6 months [72]. The outcomes were poor, with 30-day and 1-year mortality rates of 15.5% and 41.3% for NSTEMI and 31.4% and 51.2% for STEMI patients, respectively.

Immediate and straightforward coronary access for these patients is crucial yet precarious. Among the 137 analyzed cardiac CT scans from the Low-Risk TAVR (LRT) trial, the TAV frame extended above the left, right, or both coronary ostia in 48.2% of subjects [73]. The highest-risk configuration for coronary access, in which both the TAV frame extends above the level of a coronary ostium and there is a TAV commissural suture post in front of the coronary, was observed in 8.7% of subjects [74]. The RE-ACCESS study constructed a multivariate model to predict the risk of unsuccessful coronary cannulation after TAVR, combining the following three variables: (1) Evolut TAV, (2) a higher TAV-sinus of Valsalva relation, and (3) implantation depth (AUC: 0.94) [74].

### 4.4. Commissural Alignment: Does It Really Matter?

Optimizing the orientation of bioprosthetic aortic valves during SAVR is usually straightforward. The procedure involves excising calcified leaflets, followed by debridement of the aortic annulus. Subsequently, the prosthesis is implanted in an anatomically correct orientation. When the coronary ostia are positioned normally, separated by 120°, this orientation allows the surgeon to achieve the maximum distance between the ostia and valve posts. However, in the presence of aberrant coronary ostia, such as a 180° separation, offsetting the valve orientation from the norm may be necessary [75].

Conversely, in real-life TAVR practice, commissural alignment rarely occurs, and TAVs are typically deployed randomly [76]. Computational analysis studies have demonstrated that malalignment is accompanied by biomechanical disadvantages [77]. The von Mises stress (a predictor of material yield under complex loads) on the leaflets of TAVs increases as the degree of malalignment rises from 0° to 60°. A measured maximum von Mises stress increase of 37% could potentially accelerate the deterioration of the prosthesis and decrease its lifespan [77].

Moreover, it has been shown that moderate (30–45°) or severe (45–60°) commissural misalignment increases the risk of mild central aortic regurgitation after TAVR by up to sevenfold (7.8%) [76]. Additionally, commissural misalignment, particularly severe misalignment, is reportedly associated with subclinical leaflet thrombosis [78]. Further research is necessary to determine the long-term impact of commissural misalignment on TAVR durability and patients’ overall prognosis, particularly as the technique becomes more widely used in young patients.

### 4.5. TAVR Fervor: A Blindspot for Coexisting Valve Pathologies?

When AS is not the sole valve pathology, therapeutic decision-making becomes more intricate. Multiple valvular disease is not rare. Significant (i.e., moderate–severe) mitral regurgitation (MR) coexists in approximately 15% to up to one-third of AS patients [79]. Mitral stenosis (MS) or tricuspid regurgitation (TR) may also coexist with AS, but less frequently.

Notably, significant MR has been linked to a 30–50% increase in short- and mid-term mortality following TAVR [79,80,81]. Nevertheless, unlike surgery, TAVR often leaves MR untreated, with the expectation that its severity might decrease post-procedure. This assumption holds true in about half of the cases [79,80,81]. Unfortunately, the remaining patients with persistent MR ≥ 3+ exhibit significantly worse 2-year survival compared to those with MR improvement to ≤2 + (54.1% vs. 74.0%; HR 2.02 [95% CI 1.43–2.86]; and log-rank *p* = 0.007) [79]. Mauri et al. found that patients with severe (4+) MR, extensive mitral calcification, and small (≤32 mm) annular dimensions have a lower probability of regression to MR ≤ 2+ post-TAVR [79]. Furthermore, patients with structural alterations (e.g., flail leaflet, prolapse, and perforation) show no improvement, with a probability of MR ≤ 2+ being zero.

Similar observations regarding the impact of concomitant valve pathologies on overall patient prognosis have also been made in patients with severe MS [82] and significant TR [83].

Consequently, considering concomitant valvular pathology and expected longevity is critical when dealing with younger patients with AS. Heart Teams evaluating TAVR in low- and intermediate-risk patients must take these factors into account during decision making.

## 5. Lifetime Management

### 5.1. A Range of Clinical Strategies—TAVR-First Strategy for All: Are We There Yet?

In 2019, TAVR procedures surpassed SAVR in the US for the first time, coinciding with the FDA’s approval of TAVR usage in young (<65 years old) and low-risk patients [3]. TAVR’s popularity has sparked suggestions of adopting it as the index procedure for low-risk, young patients with a long life expectancy. In such a theoretically plausible lifetime scenario involving TAVR-SAVR-TAVR, patients experiencing TAV prosthesis failure would undergo a once-in-a-lifetime surgical intervention through an unexplored chest. Then, should they outlive their surgical bioprosthesis, a TAV-in-SAV procedure, proven feasible and safe in most cases, could be pursued.

However, thoroughly assessing the factors associated with this therapeutic strategy is crucial. TAVR’s applicability to young patients remains insufficiently investigated. Even in the TAVR low-risk trials (PARTNER-3 and Evolut Low-Risk), the mean patient age was 73 and 74, respectively [84,85]. The most comprehensive relevant data to date come from the NOTION trial, which, eight years after the randomization of low-risk patients to SAVR versus TAVR, demonstrated no significant differences in all-cause mortality, stroke, or myocardial infarction between the two approaches [17]. The risk of bioprosthetic valve failure was also comparable. However, both groups’ average age in this trial was also very high (79 years), resulting in a limited long-term survival rate among the initial patient cohort. Consequently, detecting SVD in TAVR patients may be obscured by high competing mortality risks, leading to potential selection bias and impeding a comprehensive assessment of TAV performance and durability. Extrapolating mid-term outcomes from these trials to younger patient cohorts warrants extreme caution. Given TAVR’s elevated rates of permanent pacemaker implantation, paravalvular leak, and heart failure hospitalization compared to SAVR, the potential adverse impact of index TAVR on long-term prognosis necessitates consideration.

Furthermore, TAVR has not been subject to investigation in randomized control trials concerning patients with bicuspid aortic valve (BAV) stenosis, a condition frequently encountered in individuals under the age of 65. Observational data reveal a significantly higher stroke rate following TAVR in this specific patient cohort when compared to SAVR. Finally, it is important to note that surgery performed after TAV failure, despite being conducted through an unexplored chest, should still be regarded as reoperative aortic root surgery. As discussed in Section 4.1, the process of TAV explantation may be technically challenging and associated with excessive operative mortality, reaching up to 20%.

The more remote scenario of TAVR-TAVR-TAVR, while also theoretically feasible, presents the full array of limitations discussed in the preceding paragraphs. These limitations include heightened risks of PPM and PVL, the need for pacemaker implantation, uncertain long-term durability, potential coronary obstruction, restricted future coronary access, and a substantially elevated operative risk if TAV(s) explantation becomes necessary.

Another critical consideration in this last scenario involves the increased thrombotic potential arising from the cumulative hardware in the aortic root. In the context of ViV procedures, the geometric constraints imposed by the leaflets and frame of the degenerated bioprosthesis result in prolonged blood residence time on the leaflets, acting as a permissive factor in leaflet thrombosis development, as demonstrated in a computational model [86]. The VIVID Registry reported a clinical valve thrombosis incidence of 7.6%, diagnosed at a median time of 101 days post-procedure [87]. In a prospective observational TAVR registry, early hypoattenuated leaflet thickening (HALT) was diagnosed in 16.0% of 804 patients. Although not associated with mortality or cerebrovascular events, HALT was strongly related to symptomatic hemodynamic valve deterioration (HR: 6.10; 95% CI: 2.59–14.29; and *p* < 0.001) [88]. In a hypothetical TAV-in-TAV-in-TAV configuration, aside from the incremental reduction in the effective orifice area with each implanted TAV, the risk of thrombosis remains unknown but could theoretically be exponentially elevated.

Given these concerns, a “TAVR first” strategy might be considered in patients with a large annulus, wide sinuses of Valsalva, and accommodating coronary ostia anatomy. Nevertheless, it is essential to acknowledge that considerable drawbacks persist, rendering this approach unsupported with current evidence and far from ideal before further investigation of TAVR in the low-risk, young patient population determines its efficacy and long-term impact on prognosis.

The “surgery first” approach, whether in the setting of a SAVR-SAVR-TAVR or SAVR-TAVR-TAVR scenario, contingent upon the parameters discussed in Section 3, remains the gold standard for managing severe AS in low-risk patients below 75 years of age (according to the European guidelines [89]) and particularly those below 65 years, as stipulated by the American Guidelines as well [90].

The SAVR first strategy capitalizes on conducting surgery at a younger age, consequently bearing an exceedingly low risk of operative mortality and morbidity, while concurrently circumventing the aforementioned risks associated with TAVR. Nonetheless, meticulous attention must be directed toward the procedural characteristics and the choice of surgical bioprosthesis for implantation, as these factors significantly impact the patient’s long-term therapeutic management and prognosis. Concomitant cardiac diseases must also be adequately addressed. The significance of hemodynamics on the durability and long-term outcomes of bioprostheses has been underscored in Section 2.2, advocating for careful consideration of liberal aortic root enlargement during the initial SAVR, as elaborated in Section 2.3.

Moreover, it is crucial to recognize that not all bioprosthetic valves are identical, regarding their durability and their potential in a future TAV-in-SAV scenario. The Inspiris Resilia valve exhibits certain advantages in this context, owing to its distinctive features, briefly presented in Section 2.2.1. Although experimental and mid-term clinical data regarding its durability are promising, long-term outcomes are eagerly awaited before definitive conclusions can be drawn.

### 5.2. Concluding Remarks

Due to their minimally invasive nature, TAVR procedures are appealing even to younger, low-risk patients with considerable life expectancies. However, it is important to acknowledge that comparative data between TAVR and SAVR are limited to a follow-up period of only 5–8 years, thereby leaving numerous uncertainties about long-term outcomes. In light of this, the involvement of Heart Teams is crucial in evaluating all available evidence and devising a comprehensive management plan for each patient that extends beyond the first 10–15 years after the index procedure. It is imperative to consider individual anatomical differences, lifestyle factors, and patient preferences rather than adopting a “one-size-fits-all” approach. The initial intervention choice has significant implications for the patient’s entire lifespan, and thus, present and cumulative risks should be carefully weighed. Based on current evidence, younger patients, particularly those under 65, should strongly consider an initial SAVR.

Future data on novel surgical bioprostheses, TAV durability, SVD, TAV explantation, and the limitations of ViV procedures will likely influence lifetime management strategies for aortic stenosis.

## Data Availability

Not applicable.
